# The ameliorative effect of ascorbic acid and *Ginkgo biloba* on learning and memory deficits associated with fluoride exposure

**DOI:** 10.2478/intox-2013-0032

**Published:** 2013-12

**Authors:** Jetti Raghu, Vasudeva C. Raghuveer, Mallikarjuna C. Rao, Nagabhooshana S. Somayaji, Prakash B. Babu

**Affiliations:** 1 Department of Anatomy, Melaka Manipal Medical College, Manipal University, Manipal, Karnataka, India; 2 Department of Pathology, Yenepoya Medical College, Yenepoya University, Mangalore, Karnataka, India; 3 Department of Pharmacology, Manipal College of Pharmaceutical Sciences, Manipal University, Manipal, Karnataka, India; 4 Department of Anatomy, Kasturba Medical College, Manipal University, Manipal, Karnataka, India

**Keywords:** fluoride, ascorbic acid, *Ginkgo biloba*, learning and memory deficits

## Abstract

Chronic exposure to fluoride causes dental and skeletal fluorosis. Fluoride exposure is also detrimental to soft tissues and organs. The present study aimed at evaluation of the effect of *Ginkgo biloba* and ascorbic acid on learning and memory deficits caused by fluoride exposure. Male Wistar rats were divided into five groups (n=6). Group 1 control. Groups 2 to 5 received 100 ppm of sodium fluoride over 30 days. Groups 3, 4 and 5 were further treated for 15 days receiving respectively 1% gum acacia solution, 100 mg/kg body weight ascorbic acid, and 100mg/kg body weight *Ginkgo biloba* extract. After 45 days, all animals were subjected to behavioural tests. The results showed that fluoride affected learning and memory. Fluoride causes oxidative stress and neurodegeneration, thereby affecting learning and memory. Ascorbic acid and *Ginkgo biloba* were found to augment the reversal of learning and memory deficits caused by fluoride ingestion.

## Introduction

Fluorine is a highly reactive gas, which combines with other ions and forms fluoride. Fluoride is the major ground water pollutant in developing countries. Phosphate fertilizers, industrial waste and combusted coal are chief sources of fluoride (Farooqi *et al.*, 2009). In the past, fluoride was thought to play a vital role in the development of the enamel of teeth and to prevent caries. In the recent view, however, fluoride is no longer considered to be needed by the body. The normal limit of fluoride to prevent caries or for the development of enamel of the teeth is 0.5 ppm (Chouhan & Flora, 2010). Excess intake of fluoride is detrimental to the bone and enamel of the teeth and results in skeletal and dental fluorosis. Chronic exposure to high doses of fluoride causes changes in the structure and function of skeletal muscle, brain and spinal cord (Shashi *et al.*, 1992). Various studies in China proved that there was a correlation between fluoride exposure and intelligence, the intelligence quotient being reduced in children with fluoride exposure (Lu Y *et al.*, 2000; Li *et al.*, 1995; Zhao *et al.*, 1996). Fluoride crosses the placental barrier, reaches the brain of offspring and causes damage to developing neurons (Du *et al.*, 2008). Fluoride can cross the blood-brain barrier, accumulate in the brain and damage the neural architecture (Geeraerts *et al.*, 1986; Mullenix *et al.*, 1995; Vani & Reddy, 2000). In addition to intelligence, learning and memory, fluoride also causes impairment in locomotor activity (Gupta *et al.*, 1993; Paul *et al.*, 1998). Chronic fluoride administration leads to altered neuronal activity and abnormal behavioral patterns (Mullenix *et al.*, 1995). Moderate consumption of fluoride has detrimental effects on learning and memory (Chioca *et al.*, 2008). Despite various defluoridation techniques available in practice, there is no single technique that would be effective to completely remove the fluoride from drinking water. Thus the magnitude of the fluorosis problem keeps increasing every year. Several researchers have tested different natural compounds like curcumin, aloe vera, oscimum sanctum, *etc.* for their efficacy against fluoride toxicity.

Neuronal function and synaptic plasticity are influenced by a number of dietary factors (Gomez-Pinilla, 2008). Ascorbic acid, a water-soluble potent antioxidant acts by scavenging free radicals, reactive oxygen species and by inhibiting lipid peroxidation (Halliwell, 1996; Halliwell, 2006). Ascorbic acid prevents oxidative stress and thus protects the brain from many neurodegenerative disorders (Harrison & May, 2009). A higher level of ascorbic acid is found in the brain than any other organ (Rice, 2000). The ascorbic acid level is not uniform throughout the brain, being highest in the amygdala, hippocampus and hypothalamus (MacGregor *et al.*, 1996). Treatment with ascorbic acid was found to reverse learning and memory deficits seen in a mouse model of Alzheimer's disease (Harrison *et al.*, 2009). Ascorbic acid prevented neurobehavioral changes caused by administration of monosodium glutamate in periadolescent rats (Narayanan *et al.*, 2010). Short-term and long-term supplementation of ascorbic acid exhibited a facilitatory effect on passive avoidance learning and memory (Shahidi *et al.*, 2008).

*Ginkgo biloba* is a Chinese medicinal plant; *Ginkgo biloba* leaf extract contains glycosides of the flavonoids kaempferol, quercetin and isorhamnetin. Further constituents are diterpene, lactones, ginkgolides A, B, C, M and J, bilobides, and biflavones ginkgetin, isoginkgetin and bilobetin (Kleijnen & Knipschild, 1992). *Ginkgo biloba* has potent effects on the central nervous system (Naik *et al.*, 2006). *Ginkgo biloba* protects neurons against ischemia, enhances cognition, preserves hippocampal mossy fibers, improves neural plasticity and prevents the cognitive deficits that result from stress or traumatic brain injury (DeFeudis & Drieu 2000). Quercetin, one of the active components of *Ginkgo biloba, was* evaluated for its mitigating role against oxidative stress in the brain caused by fluoride (Nabavi *et al.*, 2012). Behavioral impairment observed in streptozotocin treated animals was slowed down by the administration of EGb 761 (Hoyer *et al.*, 1999). Passive avoidance learning was improved in EGb 761 administerd mice compared to saline-treated and control animals (Stoll *et al.*, 1996).

Numerous studies were conducted on fluoride toxicity, however very few studies addressed the behavioral consequences of fluoride exposure. Thus the present study was undertaken also with the aim to test the efficacy of *Ginkgo biloba* and ascorbic acid against learning and memory deficits caused by fluoride ingestion.

## Material and methods

### Animals and experimental design

The experiments were conducted on one-month-old adult male rats of the Wistar strain. The animals were bred at the Manipal University, Manipal, India. The animals were reared in polypropylene cages with paddy husk as bedding material. The animals were maintained at 12:12 hour light and dark cycle, with free access to food and water. Prior approval was obtained from the institutional animal ethics committee (IAEC/KMC/21/2009–2010). All the experiments were conducted according to the guidelines given by the CPCSEA Government of India.

The animals were divided into five groups of six animals each (n=6). Group I (Control) animals had free access to ordinary tap water at fluoride level 0.5 ppm. Group II (Fluoride) group of animals had access to fluoridated water at the dose of 100 ppm for a period of 30 days. Then the animals were allowed a recovery period of 15 days. Group III (Fluoride+Vehicle) received 100 ppm of fluoride water for 30 days followed by 1% Gum acacia solution at the dose of 100mg/kg body weight for 15 days. Group IV (Fluoride+ Ascorbic Acid) animals received initially 100 ppm of fluoride water for 30 days and ascorbic acid 100 mg/kg body weight for further 15 days. Group V (Fluoride+*Ginkgo biloba*) received 100 ppm fluoride water for the initial 30 days followed by *Ginkgo biloba* at the dose of 100 mg/kg body weight for 15 days. At the end of 45 days, all the groups of animals were subjected to the T-maze and passive avoidance behavioral tests.

### Administration of sodium fluoride

Sodium fluoride was obtained from the Nice Chemicals Cochin India. Sodium fluoride (221 mg) was dissolved in one liter of distilled water to achieve 100 ppm of fluoride. Fluoride was administered as drinking water.

### Administration of *Ginkgo biloba* and ascorbic acid

*Ginkgo biloba* and ascorbic acid were administered at the dose of 100 mg/kg body weight orally with the help of an oral feeding needle attached to the syringe. Vitamin C was obtained from the Nice Chemicals Cochin India. The *Ginko biloba* extract was purchased from the Reindeer Biotech co Ltd. China.

### Behavioral tests

At the end of the experimental period, the animals were subjected to the T-maze and Passive avoidance tests to assess spatial learning and memory, respectively. As rodents are nocturnal animals, all the behavioral tests were conducted in a dark room in a silent environment during the night between 7 p.m.–10 p.m.

### T-maze test

This includes the spontaneous alternation test and rewarded alternation test. The method described by Dunnett *et al.* (1982) was followed.

### Passive avoidance test

The procedure described by Bures *et al.* (1983) was followed.

### Statistical analysis

Data were analyzed using one-way anova followed by Bonferroni's post-test using Graph Pad Prism, version 5 (Graph Pad Prism Software Inc., USA).

## Results

### T-maze test

The results of the T-maze test are represented in Table 1.

**Table 1:** The results of T-maze test.

Groups	n	T-MAZE TEST		
		Spontaneous alternation test		Rewarded alternation test
		Number of alternations	% bias	% of correct response
C	6	15.67±1.75	52.77±4.30	75.69±4.87
F	6	8.83±1.16***	69.44±3.40***	55.55±4.30***
F+V	6	10.50±1.51	66.66±3.72	56.25±4.37
F+C	6	14.17±0.75$$$	58.33±3.73$$$	67.36±3.13$$$
F+GB	6	13.83±1.32###	56.25±4.37###	66.66±3.72###

Each value represents Mean ± SD.

*** C vs. F: p<0.001

$$$ F vs. F+C: p<0.001

### F vs. F+GB: p<0.001 (One way ANOVA, Bonferroni's test).

C: Control; F: Fluoride; F+V: Fluoride + Vehicle; F+C: Fluoride + Vitamin C; F+GB: Fluoride + *Ginkgo biloba*

#### Effect of fluoride on spatial learning

Animals that received fluoride showed significant impairment in spatial learning, in the form of more percentage bias, less alternations and less percentage of correct response in comparison with control animals which received ordinary tap water.

#### Effect of ascorbic acid on spatial learning of fluoride treated animals

Ascorbic acid treated animals showed significant improvement in the spatial learning deficits in comparison with the fluoride group of animals. From the results of the present study it is evident that ascorbic acid can protect the brain from several neurotoxins, especially fluoride.

#### Effect of Ginkgo biloba on spatial learning in animals that received fluoride

The *Ginkgo biloba* treated group of animals showed more alternations, lower percentage bias and higher percentage correct response compared to the fluoride animals.

### Passive avoidance test

The results of the passive avoidance test are presented in Table 2.

**Table 2:** The results of Passive avoidance test.

Groups	n	PASSIVE AVOIDANCE TEST			
		Exploration		Retention	
		Tot. time in small compartment (sec)	No. of crossings	Tot. time in small compartment (sec)	No. of crossings
C	6	392.5±20.4	12.17±1.47	135.7±15.6	7.00±1.41
F	6	398.3±13.6	11.83±1.47	250.8±21.9***	13.00±1.41***
F+V	6	395.3±13.3	11.33±1.21	264.3±16.7	12.17±1.16
F+C	6	396.2±8.25	13.00±1.41	201.0±7.21$$$	8.83±1.47$$$
F+GB	6	393.3±16.3	12.83±1.16	198.3±6.05###	9.00±1.78###

Each value represents Mean ± SD.

*** C vs. F: p<0.001

$$$ F vs. F+C: p<0.001

### F vs. F+GB: p<0.001 (One way ANOVA, Bonferroni's test).

C: Control; F: Fluoride; F+V: Fluoride + Vehicle; F+C: Fluoride + Vitamin C; F+GB: Fluoride + *Ginkgo biloba*

#### Exploration

No significant differences were observed among the groups during exploration for the time spent in the dark compartment.

#### Retention: Effect of fluoride exposure on passive avoidance learning

The fluoride group of animals showed poor memory retention, spending more time in the dark compartment compared to the control group.

#### Effect of ascorbic acid on passive avoidance learning

Memory retention was significantly high in the animals that received ascorbic acid compared with the fluoride treated animals.

#### Effect of Ginkgo biloba on passive avoidance learning

The animals that received *Ginkgo biloba* showed significant high memory retention compared to the fluoride group of animals.

## Discussion

Chronic fluoride exposure results in accumulation of fluoride in the body, especially in the teeth, bones and in other soft tissues like muscles, ligaments and the brain. Several natural compounds were tested for their efficacy against fluoride toxicity. In the present study, we tested detrimental effects of fluoride exposure with special reference to spatial learning and memory; we also investigated the protective effect of *Ginkgo biloba* and ascorbic acid against the learning and memory deficits caused by the fluoride.

In the T-maze test, the fluoride group of rats showed significant learning and memory deficits compared to control rats. These deficits were however not recorded in rats treated with *Ginkgo biloba* and ascorbic acid. In the passive avoidance test, there was no significant difference in behavior between the groups during exploration, yet during the retention test, the fluoride group of rats spent more time in the small compartment, indicating memory impairment, while ascorbic acid and *Ginkgo biloba* treated rats spent significantly less time in the small compartment, suggesting a protective effect against fluoride toxicity. The results of the present study are consistent with previous studies indicating that fluoride intoxication causes anxiety, learning and memory impairment (El-lethey *et al.*, 2010). Fluoride intoxicated rats were found to have poor performance in the maze test (Basha & Sujitha, 2012). Compared to these results, in our study sodium fluoride exposed rats showed higher percentage bias and lower number of alternations, which is considered to be an index of learning and memory impairment. In a multigenerational study conducted on three successive generations of animals, fluoride caused significant learning disabilities in all three generations of animals (Basha *et al.*, 2011).

Fluoride may interfere with oxygen metabolism and generate oxygen free radicals, which are responsible for the diminished learning and memory (Chirumari & Reddy, 2007). Fluoride exposed pups took the longest time to find the hidden platform, showing that fluoride alters cognitive responses and behavior (Wu *et al.*, 2008). The learning and memory deficits are the result of interaction of fluoride with the activity of the enzyme acetylcholine esterase. Exposure to fluoride results also in a decrease of the nicotinic acetylcholine receptors (Shan *et al.*, 2004). Fluoride exposed rats showed inhibition of spontaneous motor activity, due to alterations in the function of neurotransmitters (Paul *et al.*, 1998). Moderate exposure to fluoride had harmful effects on learning and memory, caused impairment in habituation and reduced the number of avoidance responses (Chioca *et al.*, 2008). Fluoride administered in drinking water reduced the latency in the step-down test (Wu *et al.*, 2006). Impaired learning and memory ability is due to changes in the synaptic structure, with a decrease in the thickness of postsynaptic density and an increase in the width of the synaptic cleft (Zhang & Shen, 2008).

Ascorbic acid has neuromodulatory and cognition enhancing properties. Ascorbic acid may act as an acetylcholine esterase inhibitor, thus attenuating scopolamine induced spatial learning deficits (Harrison *et al.*, 2009). Ascorbic acid may facilitate cholinergic transmission and improve the cognitive impairment. Exogenous administration of ascorbic acid might result in high antioxidant levels which fight free radicals and oxidative stress caused by fluoride (Parle *et al.*, 2003). In a recent study from China, *Ginkgo biloba* extract improved the cognitive impairment caused by chronic fluorosis, as assessed by the Y maze test (Zhang *et al.*, 2013]. In the current study, Ginkgo biloba ameliorated learning and memory impairments caused by fluoride exposure. However in the current study learning and memory were assessed by using the T-maze test and improvement was observed with the dose of 100 mg/kg body weight. Treatment with *Ginkgo biloba* extract Egb761 enhanced the synaptic plasticity in the hippocampus, which in turn could ameliorate the deficits in spatial learning and memory (Wang *et al.*, 2006). Long-term administration of *Ginkgo biloba* extract improved spatial memory by altering the levels of neurotransmitters in several regions of the brain (Blecharz-Klin K *et al.*, 2009).

The results of the present study are in agreement with studies published in the field of fluoride research, showing that chronic exposure to fluoride leads to learning and memory deficits since fluoride alters the level of neurotransmitters, synaptic structure, and cholinergic mechanisms. The learning and memory deficits were however not severe in the ascorbic acid and *Ginkgo biloba* treated groups, indicating that both substances exhibited neuroprotective effects, attributed to their antioxidant and neurotropic properties.
